# A Mechanism-Oriented Multiphysics Study of Scratch-Width-Dependent Galvanic Protection Loss in Mechanically Damaged Hot-Dip Galvanized Steel Enclosures

**DOI:** 10.3390/ma19173600

**Published:** 2026-08-24

**Authors:** Junqi Mai, Wenkai Xiao, Feiyang Yu, Huijiu Wang, Junwen Wang, Limin Yang, Xiaozhuan Zhang, Lin Gui, Xin Lin, Shijing Wu, Xian Zhai

**Affiliations:** 1 School of Power and Mechanical Engineering, Wuhan University, Wuhan 430072, China; 2023302081085@whu.edu.cn (J.M.); xiaowenkai2003@163.com (W.X.); 2019302080337@whu.edu.cn (F.Y.); wsj@whu.edu.cn (S.W.); 2 Bull Group Co., Ltd., Ningbo 315000, China

**Keywords:** mechanical damage, hot-dip galvanized steel, galvanic protection, ohmic drop, scratch width, multiphysics simulation

## Abstract

Mechanical scratches that penetrate the protective layers expose the steel substrate and establish a zinc–steel galvanic couple beneath an electrolyte film. This study develops a mechanism-oriented multiphysics model of scratch-width-dependent galvanic protection loss in hot-dip galvanized (HDG) steel enclosures. The model couples Butler–Volmer interfacial kinetics with equilibrium potentials related through the Nernst framework, Nernst–Planck transport of Zn^2+^, OH^−^, Na^+^, Cl^−^, and dissolved O_2_, electrolyte charge conservation, reaction-derived boundary fluxes, a simplified corrosion-product deposition and transport-resistance treatment, and level-set tracking of interface evolution. Numerical field cases at scratch widths of 1, 3, and 5 mm show that widening the scratch increases the exposed-steel cathodic demand and lengthens the ionic-current path from the zinc edges to the scratch center. The resulting ohmic drop reduces the protective current and permits local iron dissolution when the center can no longer be maintained at a sufficiently negative potential. NSS morphology, cross-sectional SEM/EDS, corrosion-depth trends, and XRD observations show qualitative consistency with this mechanism. Under the investigated NSS conditions, a marked descriptive change occurs between the experimentally sampled widths of 2 and 3 mm, with 3 mm representing the first sampled condition showing pronounced protection loss. Because replicate-level corrosion-depth data and independent electrochemical measurements are unavailable, no statistical significance or quantitative model validation is claimed.

## 1. Introduction

Driven by the rapid expansion of the new energy vehicle industry, DC charging piles are being deployed outdoors at increasing density. As the first protective barrier of the equipment, the enclosure must withstand both mechanical impact and complex corrosive environments. Owing to the combined protection provided by its physical barrier and cathodic-protection functions, hot-dip galvanized (HDG) steel has become a mainstream material for charging-pile enclosures [1,2]. In current industrial practice, HDG steel sheets are commonly combined with polymer coatings to form a composite protective system intended to maintain a service life of more than eight years in C4 corrosive environments [3,4]. However, scratches, impacts, and other forms of mechanical damage can readily occur during transportation, hoisting, installation, commissioning, and daily use. Once the zinc layer is damaged, the steel substrate is exposed to corrosive media, creating initiation sites for localized corrosion. The coupling of mechanical damage and corrosive media often accelerates localized corrosion, causes premature failure of the protective system, and threatens the operational safety and service life of the charging equipment [5,6,7].

To date, studies on corrosion failure of HDG steel have mainly focused on coating-process optimization and interface control [8,9,10], the intrinsic corrosion resistance of coatings and the effects of alloying elements [11,12,13,14,15], corrosion-product evolution and barrier effects [16,17], and environmental or stress-assisted degradation [18,19]. Corrosion failure after a through-scratch remains less clearly resolved because electrochemical kinetics, ionic transport, geometry, and interface evolution interact nonlinearly. The 1.2 mm and 1.5 mm sheets examined here are two project-supplied commercial enclosure gauges sourced in Wuhan, China; comparing them provides an initial assessment of whether substrate thickness changes the local protection transition without treating either gauge as a universal industry optimum. The central question is therefore how scratch width changes the ability of the surrounding zinc to cathodically protect the exposed steel.

Multiphysics simulation provides a means to separate the coupled roles of interfacial kinetics, electrolyte transport, potential distribution, and evolving geometry in a damaged zinc–steel system [20,21,22,23]. Accordingly, this study uses numerical results to explain why increasing scratch width causes loss of galvanic protection, and uses salt-spray morphology, corrosion-depth trends, SEM/EDS, and XRD observations as qualitative support for the proposed mechanism. The experimentally observed transition is reported only for the material and exposure conditions investigated here.

## 2. Simulation Model Construction

COMSOL Multiphysics 5.6 (COMSOL AB, Stockholm, Sweden) was used to model a through-scratch zinc–steel couple under a continuous electrolyte film. It couples Nernst–Planck multi-species transport, Butler–Volmer interfacial kinetics with Nernst equilibrium potentials, electrolyte charge conservation, reaction-derived fluxes, simplified corrosion-product deposition and transport resistance, and level-set interface tracking. The field cases use scratch widths of 1, 3, and 5 mm to identify the physical origin of scratch-width-dependent protection loss. They provide mechanism-oriented comparisons rather than calibrated predictions of absolute potential, current, corrosion depth, coating life, or a universal critical width.

### 2.1. Physical Representation and Model Assumptions

The model represents a two-dimensional cross-section through a machined scratch that penetrates the protective layers and exposes the steel substrate. The experimental specimens have a measured zinc-coating thickness of 8 ± 2 µm, whereas the model retains an enlarged 100 µm computational zinc domain. The 100 µm value is a numerical-domain choice used to keep the moving Zn/electrolyte interface resolvable during the level-set calculation; it is not intended to reproduce the physical coating thickness. Because a zinc-thickness sensitivity study is not available, the influence of this idealization on modeled zinc inventory and local anode/cathode geometry remains a limitation. Accordingly, the model is interpreted through relative spatial distributions and scratch-width trends, not one-to-one values of current, interface recession, corrosion depth, lifetime, or threshold width. Only the exposed zinc edges, exposed steel, and overlying electrolyte participate in the electrochemical domain. The intact polymer-coated region outside the damage is treated as an insulating, zero-current, and zero-species-flux boundary, so the conclusions do not apply to shallow scratches that leave the steel unexposed.

The salt-spray electrolyte is idealized as a stationary continuous liquid film over the through-scratch region. The model resolves local galvanic coupling, ionic transport, electrolyte-potential distribution, ohmic drop, reaction-derived boundary fluxes, interface evolution, and the influence of the simplified corrosion-product deposition and transport-resistance treatment. Random droplet deposition, complete wet–dry cycling, local film rupture, and three-dimensional morphology remain outside the model scope.

### 2.2. Electrochemical Reaction Network and Interfacial Kinetics

A through-scratch establishes a galvanic couple in which zinc, having the more negative potential, dissolves anodically and supplies electrons through the metal to the exposed steel. Under neutral aerated conditions, oxygen reduction on the steel consumes those electrons and generates OH^−^. The local Butler–Volmer currents in Table 1 are converted through stoichiometry into Zn^2+^, O_2_, and OH^−^ boundary fluxes, while Zn^2+^, OH^−^, Na^+^, Cl^−^, and dissolved O_2_ are transported through the electrolyte. Corrosion-product effects are represented through a simplified functional deposition/transport-resistance treatment rather than a fully parameterized porous-medium formulation. As scratch width increases, the exposed-steel cathodic area and current demand increase, the electrolyte path from each zinc edge to the center lengthens, and the associated ohmic drop reduces the protective current. Local iron dissolution is introduced when the steel becomes insufficiently protected; Fe^2+^ transport and its concentration-dependent equilibrium-potential correction are outside the present mechanism-oriented formulation.

The equilibrium potential is evaluated within a generalized Nernst framework using the standard-potential inputs listed in Table 2b. The resulting equilibrium-potential fields are interpreted comparatively; reaction-specific activity corrections are not used to support absolute electrochemical prediction. The relationship between the local current density i and the overpotential η is then described by the Butler–Volmer equation:

(1) $$i=i_{0}\{exp(\frac{\alpha_{a}nF\eta }{RT})-exp(\frac{-\alpha_{c}nF\eta }{RT})\}$$

where i_0_ is the exchange current density; α_a_ and α_c_ are the anodic and cathodic charge-transfer coefficients, respectively; n is the number of electrons transferred; F is the Faraday constant; R is the universal gas constant; and T is the absolute temperature. The overpotential is η = φ_s_ − φ_l_ − E_eq_, where φ_s_ and φ_l_ are the electrode and electrolyte potentials and E_eq_ is the equilibrium potential related to the local electrochemical state through the Nernst framework.

### 2.3. Governing Equations and Multiphysics Coupling

(1) Species Transport and Mass Conservation

The transport of Zn^2+^, OH^−^, Na^+^, and Cl^−^ in the electrolyte is governed by the Nernst–Planck equation; dissolved O_2_ is treated as a neutral diffusing species under the same conservation framework:

(2) $$N_{i}=-D_{i}\nabla c_{i}-z_{i}u_{i}Fc_{i}\nabla \varphi_{l}$$

where N_i_ is the flux of species i, and c_i_, D_i_, z_i_, and u_i_ denote its concentration, diffusion coefficient, charge number, and ionic mobility, respectively.

The ionic mobility is related to the diffusion coefficient through the Nernst–Einstein relation, *u_i_* = *D_i_*/(*RT*).

The spatiotemporal variation in each species concentration follows mass conservation:

(3) $$\frac{\partial c_{i}}{\partial t}+\nabla \cdot N_{i}=0$$

A common dissolved-oxygen boundary condition was applied to all modeled scratch widths. Because this condition was not independently calibrated to the NSS specimens, oxygen-related results are used only for relative mechanistic comparison rather than absolute oxygen-consumption or cathodic-current prediction.

(2) Electrolyte Potential and Charge Conservation

Assuming electroneutrality and interfacial localization of all charge-transfer reactions, electrolyte charge conservation is written as:

(4) $$\nabla \cdot i_{l}=0$$

The total electrolyte current density is obtained by summing the charge carried by all ionic-species fluxes:

(5) $$i_{l}=F\sum_{k}z_{k}N_{k}$$

where F is the Faraday constant and N_k_ is the flux of species k, expressed as:

(6) $$N_{k}=-D_{k}\nabla c_{k}-\frac{z_{k}D_{k}F}{RT}c_{k}\nabla \varphi_{l}+c_{k}u$$

where D_k_ is the diffusion coefficient, c_k_ is concentration, z_k_ is charge number, φ_l_ is electrolyte potential, and u is macroscopic electrolyte velocity. The present model adopts u = 0, so transport comprises diffusion and electromigration without convection.

The electrolyte current density is represented in conductivity form as:

(7) $$i_{l}=-\kappa \nabla \varphi_{l}$$

where κ is the effective electrolyte conductivity. It is treated as a constant value of 8.0 S/m in the present model (Table 2a).

Substitution into charge conservation gives the conductivity-form charge-conservation equation:

(8) $$\nabla \cdot (-\kappa \nabla \varphi_{l})=0$$

κ is treated as a constant effective conductivity in the present model; under this assumption, Equation (8) reduces to ∇^2^φ_L_ = 0.

Boundary conditions are −n·i_l_ = j_loc_ at the electrode surface and n·i_l_ = 0 at insulating boundaries.

(3) Faradaic Interface Evolution

A level-set function ψ is used to track interface evolution, with its zero level set representing the corrosion interface. Its advection equation is:

(9) $$\frac{\partial \psi }{\partial t}+v_{n}|\nabla \psi |=0$$

where v_n_ is the interface-normal velocity. Faraday’s law gives:

(10) $$v_{n}=\frac{j_{n}M}{nF\rho }$$

where M and ρ are the molar mass and density of the dissolving metal, respectively, and j_n_ is the corresponding normal Faradaic current density. Substituting v_n_ into the level-set equation couples electrochemical dissolution to interface motion.

(4) Simplified Corrosion-Product Treatment

The present formulation represents corrosion-product effects through a simplified functional deposition/transport-resistance treatment rather than a fully parameterized porous-product layer. No independently calibrated deposition/resistance coefficients are used for quantitative prediction. This component is therefore interpreted only through its qualitative influence on concentration, potential, and current redistribution.

### 2.4. Geometry, Boundary Conditions, and Numerical Implementation

**Figure 1:**
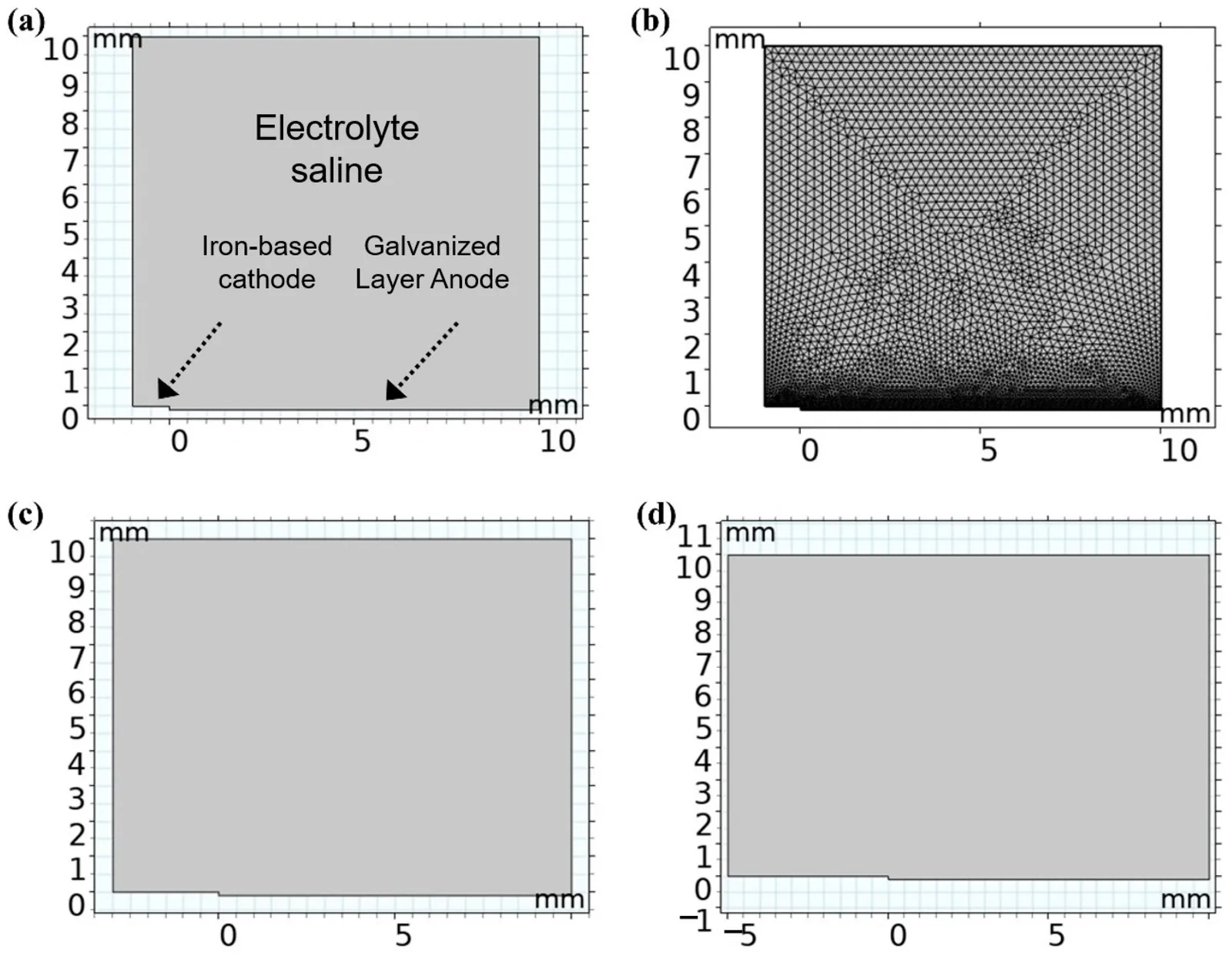
Geometry and mesh of the HDG steel bimetallic system under mechanical damage: (**a**) 1 mm scratch model; (**b**) mesh of the 1 mm scratch model; (**c**) 3 mm scratch model; and (**d**) 5 mm scratch model. The schematic zinc-domain thickness is not drawn to the experimental coating-thickness scale.

A two-dimensional cross-sectional model of the through-scratch HDG steel system was established (Figure 1). The experimental specimens had a measured zinc-coating thickness of 8 ± 2 µm, whereas the model uses an enlarged 100 µm computational zinc domain to maintain a resolvable moving interface during level-set evolution. The schematic geometry is therefore not drawn to the experimental coating-thickness scale. The scratch exposes the steel substrate and leaves zinc at both edges; the intact polymer-coated region is represented by insulating, no-flux boundaries. Field distributions were evaluated for 1, 3, and 5 mm scratches. At the open boundary, supporting species use ci = ci,bulk, with the Na^+^ and Cl^−^ values listed in Table 2a. Species concentrations were initialized uniformly from these bulk supporting-ion conditions. A non-uniform free triangular mesh with local refinement at metal/electrolyte interfaces was used to resolve steep current, potential, and concentration gradients. Because no separate mesh-independence study is available, the resulting fields are interpreted qualitatively rather than as mesh-verified absolute predictions.

### 2.5. Model Parameters and Parameter-Selection Rationale

Table 2a,b summarize the model geometry, environmental conditions, transport and kinetic parameters, standard potentials, boundary conditions, and physical roles used in the present formulation. The 100 µm value denotes an enlarged computational zinc-domain thickness, whereas 8 ± 2 µm is the measured specimen coating thickness. The computational thickness was not independently calibrated or subjected to a thickness-sensitivity study; uncalibrated parameters are therefore treated as comparative model inputs rather than as a basis for quantitative corrosion-depth prediction.

The reaction standard potentials E^0^ and exchange current densities define the thermodynamic reference states and kinetic scales of the Butler–Volmer implementation. The equilibrium potential is related to the local electrochemical state through a generalized Nernst framework. Because reaction-specific activity corrections are not used to support absolute predictions, the equilibrium-potential outputs are restricted to comparative interpretation.

### 2.6. Numerical Reproducibility and Interpretation Boundary

The numerical results are restricted to comparative, mechanism-oriented interpretation. The present formulation does not provide a complete reproducibility package comprising reaction-specific Nernst activity expressions, a calibrated oxygen boundary concentration, a fully parameterized product-layer resistance law, an experimental reference-electrode mapping, element-count and mesh-independence data, and complete solver, time-stepping, and convergence settings. These limitations preclude strict independent reproduction and quantitative validation. A complete reproducibility package together with zinc-thickness and mesh-sensitivity analyses is required before absolute numerical predictions can be assessed.

## 3. Experimental Methods

### 3.1. Materials and Sample Preparation

Commercial HDG steel sheets (Q/BB385-2022-DC51D) with thicknesses of 1.2 and 1.5 mm were used. The measured zinc-coating thickness was 8 ± 2 µm. The substrate composition and measured coating masses are reported separately in Table 3a,b.

Specimens measured 50 × 50 × t mm (t = 1.2 or 1.5 mm). Standardized through-scratches with width levels of 0.5, 1, 2, 3, 4, and 5 mm were machined using a controlled milling procedure. The scratch depth was sufficient to penetrate the protective layers and expose the steel substrate. Scratch dimensions were checked by SEM before exposure.

### 3.2. Neutral Salt-Spray Exposure

Neutral salt-spray (NSS) tests were performed in accordance with GB/T 10125-2021 using a 5 wt.% NaCl solution prepared from commercially sourced NaCl (Wuhan, China) at pH 6.5–7.2 and 35 ± 1 °C under continuous spraying. The exposure periods were 0, 6, 12, 18, 24, 48, 72, 96, 120, 144, 168, and 192 h. Specimens were removed at the assigned times for morphology, composition, phase, and corrosion-depth characterization.

### 3.3. Morphology and Corrosion-Product Characterization

Corrosion-depth profiles and three-dimensional morphologies were obtained using a 3D optical profilometer (NewView 9000; ZYGO, Middlefield, CT, USA). Cross-sectional morphology was observed by scanning electron microscopy (SEM; CLARA; TESCAN, Brno, Czech Republic), elemental distributions were analyzed by energy-dispersive X-ray spectroscopy (EDS; INCA250; Oxford Instruments, High Wycombe, UK), and corrosion-product phases were identified by X-ray diffraction (XRD; SmartLab SE; Rigaku, Akishima, Japan).

### 3.4. Evidence Scope and Statistical Treatment

The corrosion-depth curves are treated as descriptive observations. Specimen-level replicate data needed to establish the effective n at each width/time point, calculate standard deviations or confidence intervals, or perform inferential testing are unavailable; accordingly, no error bars or statistical significance are claimed. SEM, EDS, and profilometry are used for qualitative or descriptive corroboration only. EIS data, fitted impedance parameters, and independent electrochemical potential/current measurements are unavailable and are not used for quantitative model validation.

## 4. Results and Discussion

### 4.1. Multiphysics Coupled Analysis of the Hot-Dip Galvanized Steel Corrosion Model

#### 4.1.1. Potential and Current Density Distribution

**Figure 2:**
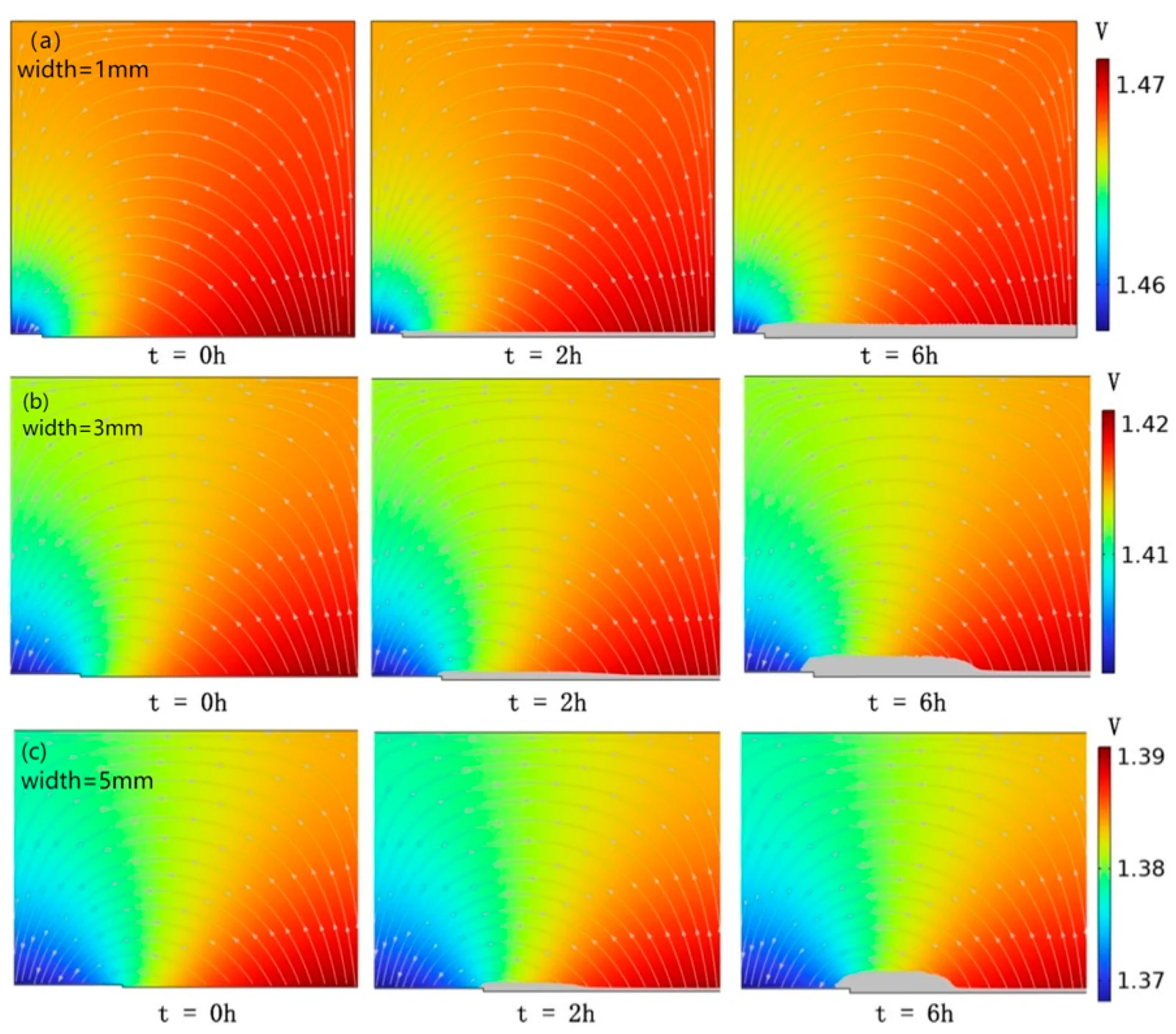
Simulated electrolyte potential distributions during the corrosion process of galvanized steel sheets with varying scratch widths: (**a**) 1 mm; (**b**) 3 mm; and (**c**) 5 mm. Arrows: current direction.

Figure 2 shows the electrolyte-potential field for the modeled 1, 3, and 5 mm scratches. The contours are interpreted in terms of spatial gradients, relative differences, and scratch-width-dependent trends. The plotted potential values use an internal numerical datum and are not compared quantitatively with an experimental reference electrode. With increasing scratch width, the broader relative potential variation is consistent with a longer ionic-current path and greater ohmic loss.

**Figure 3:**
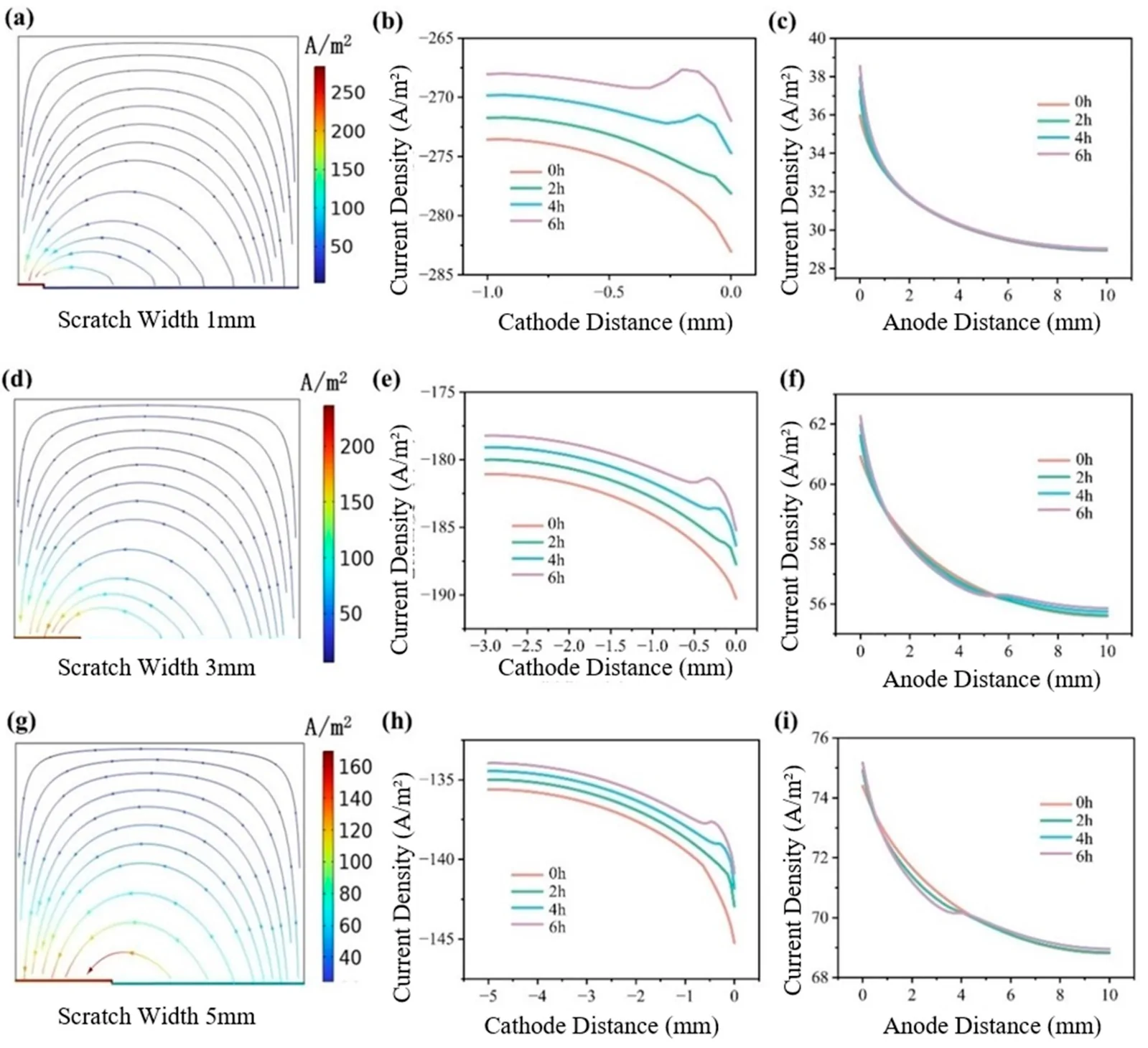
Simulated electrode current-density distributions for galvanized steel sheets with different scratch widths: (**a**–**c**) 1 mm scratch model, including current-density distribution, cathodic current density versus distance, and anodic current density versus distance; (**d**–**f**) 3 mm scratch model; and (**g**–**i**) 5 mm scratch model. Arrows: local current direction.

The current-density distribution (Figure 3) complements the potential field. Protective current is concentrated near the zinc–steel edges and decays with distance across the exposed steel. Increasing scratch width spreads the cathodic demand over a larger area while reducing the current reaching the center. In the 5 mm case, local anodic current appears on the central steel surface, marking the transition from zinc-supported cathodic protection toward local steel dissolution.

#### 4.1.2. Ion Concentration Distributions

**Figure 4:**
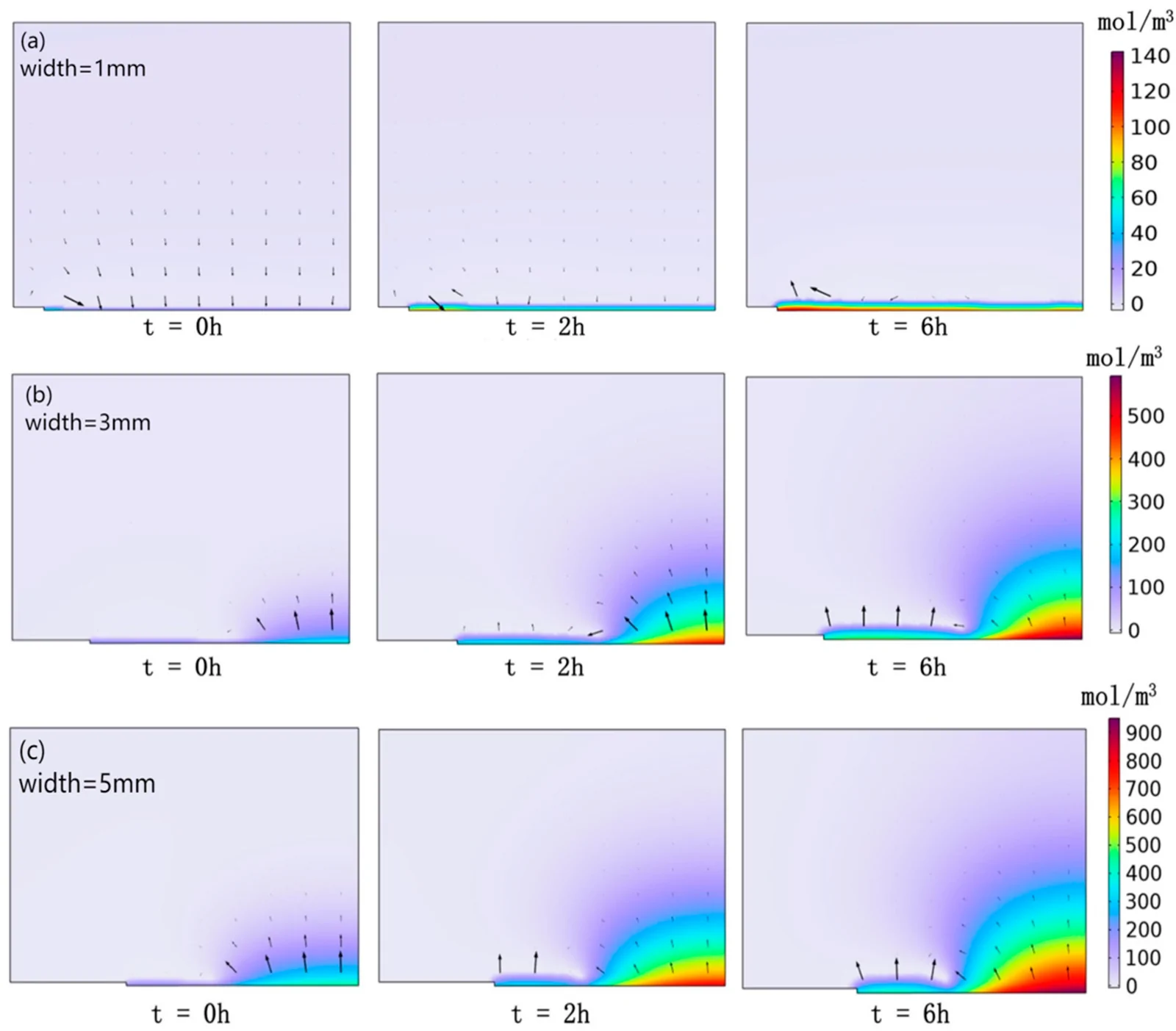
Simulated zinc ion (Zn^2+^) concentration distributions during corrosion of galvanized steel sheets with varying scratch widths: (**a**) 1 mm; (**b**) 3 mm; and (**c**) 5 mm. Arrows: Zn^2+^ flux direction.

**Figure 5:**
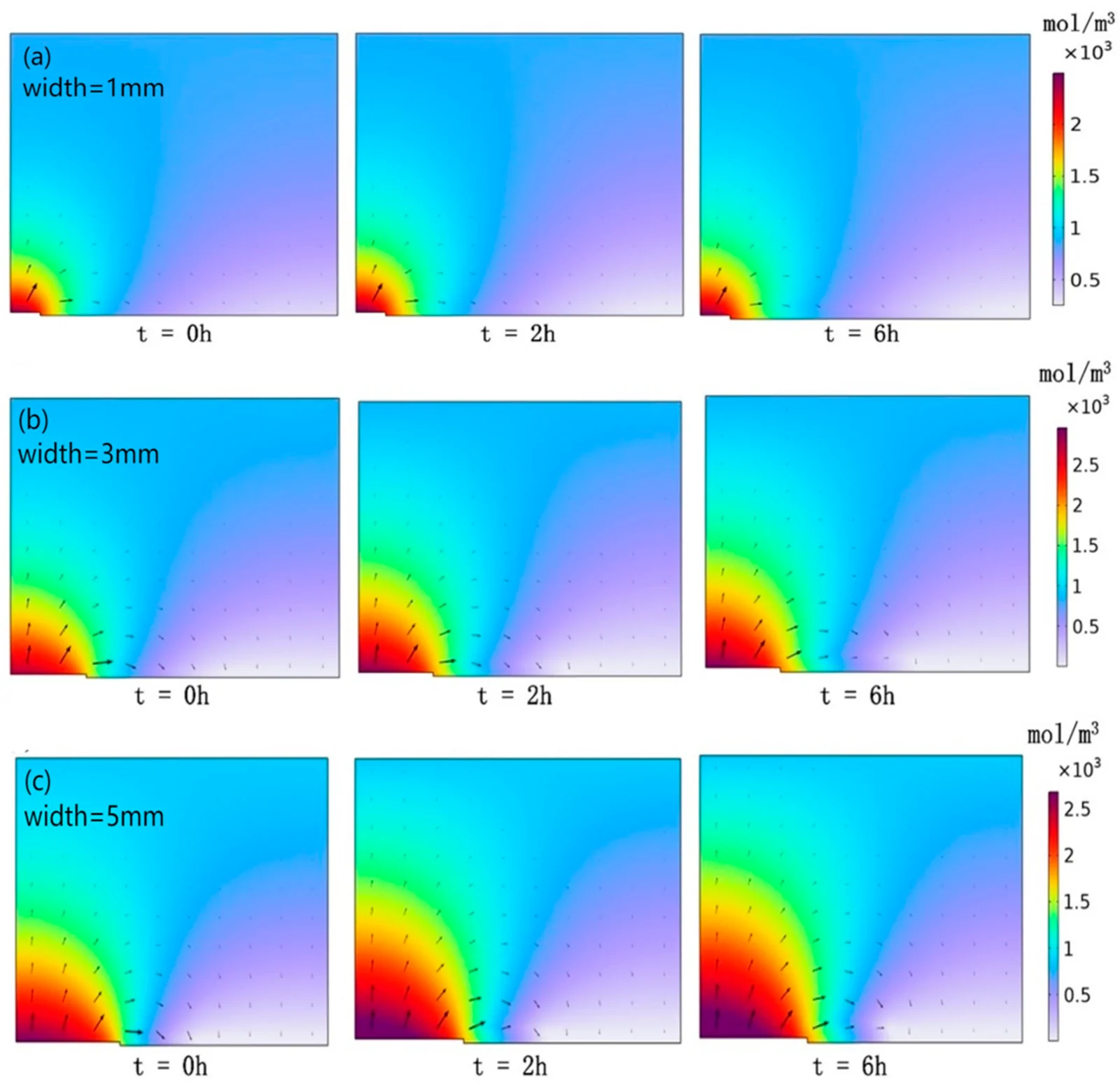
Simulated chloride ion (Cl^−^) concentration distributions during corrosion of galvanized steel sheets with varying scratch widths: (**a**) 1 mm; (**b**) 3 mm; and (**c**) 5 mm. Arrows show Cl^−^ flux.

The Zn^2+^ and Cl^−^ concentration fields are shown in Figure 4 and Figure 5. The Zn^2+^ color scales indicate peak values of approximately 140 mol/m^3^ for the 1 mm case, 500 mol/m^3^ for the 3 mm case, and 900 mol/m^3^ for the 5 mm case. The broader Zn^2+^ distribution at larger widths reflects the greater damaged area and wider transport domain; these field values are not interpreted as a corrosion-depth prediction. The simplified corrosion-product deposition and transport-resistance treatment contributes to redistribution of concentration, potential, and current. The broader Cl^−^ distribution indicates enhanced chloride transport within the damaged region; detailed chloride-complex and complete solid-phase chemistry are not independently resolved.

### 4.2. Salt-Spray Corrosion Evolution and Morphology

#### 4.2.1. Cross-Sectional Corrosion Morphology

**Figure 6:**
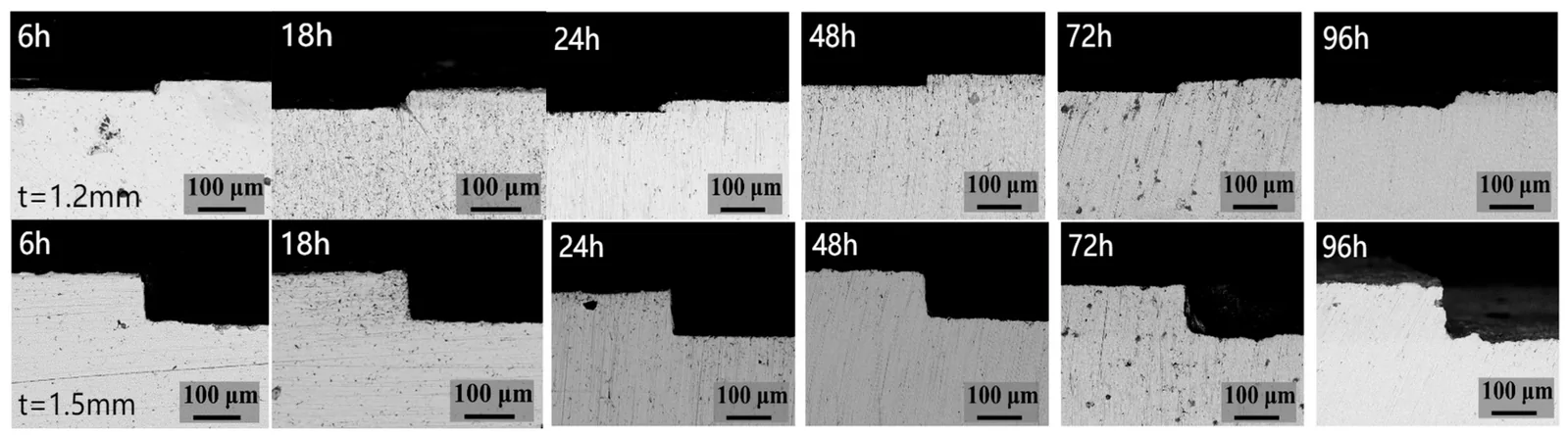
SEM images of the 1 mm scratch-width specimens (t = 1.2/1.5 mm) after different NSS exposure durations.

**Figure 7:**
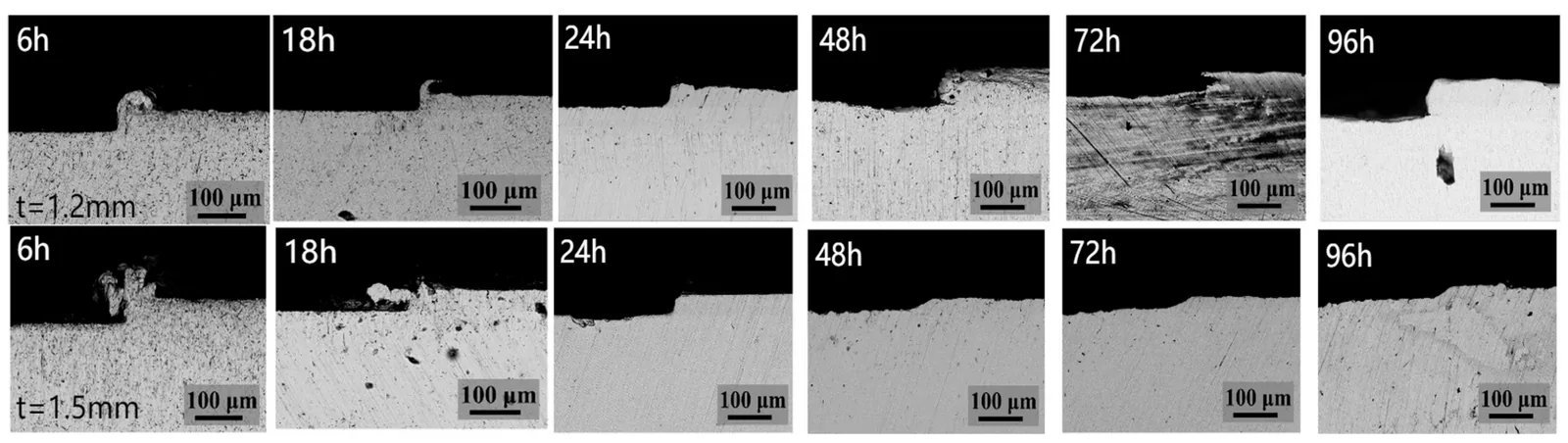
SEM images of the 2 mm scratch-width specimens (t = 1.2/1.5 mm) after different NSS exposure durations.

Cross-sectional SEM provides descriptive observations of local corrosion depth and morphology in the zinc coating and steel substrate under explicitly identified sheet gauges. The 1.2 mm and 1.5 mm specimens show broadly similar width-dependent corrosion modes. For the 1 and 2 mm scratches (Figure 6 and Figure 7), zinc dissolution at the scratch edges dominates initially and the steel remains comparatively protected. With exposure, the zinc-side grooves deepen and isolated substrate pits appear, indicating gradual attenuation of the protective current.

In the initial stage (0–24 h), dissolution of the zinc coating at the scratch edges formed grooves, while the steel substrate remained comparatively protected.

In the middle stage (48–72 h), the grooves at the scratch edges deepened and widened, and slight, uneven corrosion pits appeared on the steel substrate.

In the late stage (after 72 h), corrosion pits within the scratch deepened markedly and expanded toward both sides, producing a typical bowl-shaped cross-section.

**Figure 8:**
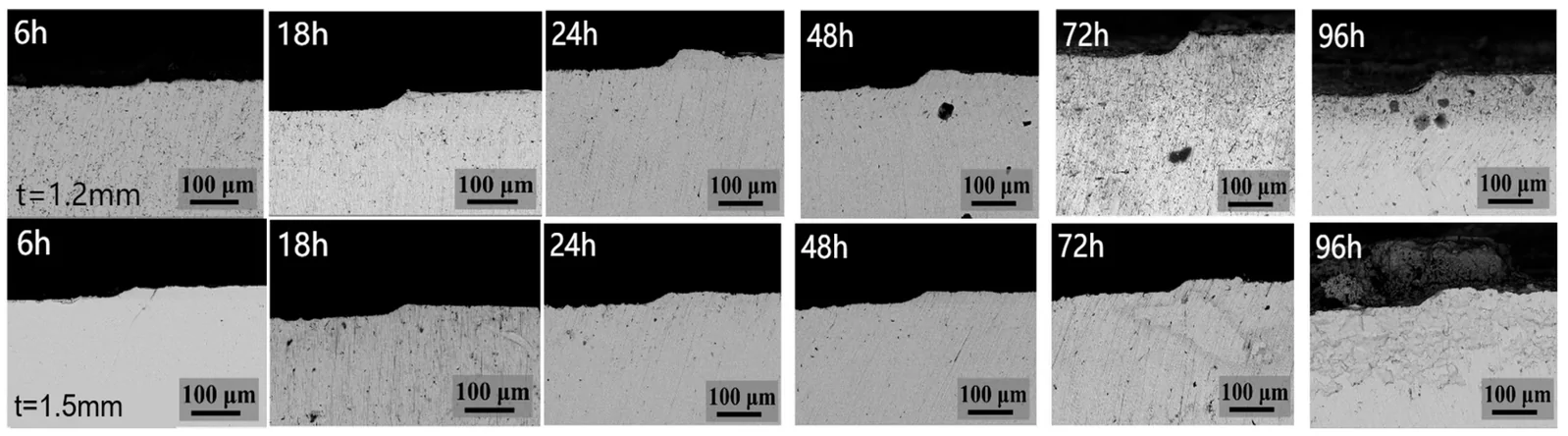
SEM images of the 3 mm scratch-width specimens (t = 1.2/1.5 mm) after different NSS exposure durations.

For the 3 mm scratch (Figure 8), widespread shallow attack appears on the steel from the early exposure stages and develops into deeper, connected damage with time. This is the first experimentally sampled width at which the center clearly shows sustained protection loss under NSS.

**Figure 9:**
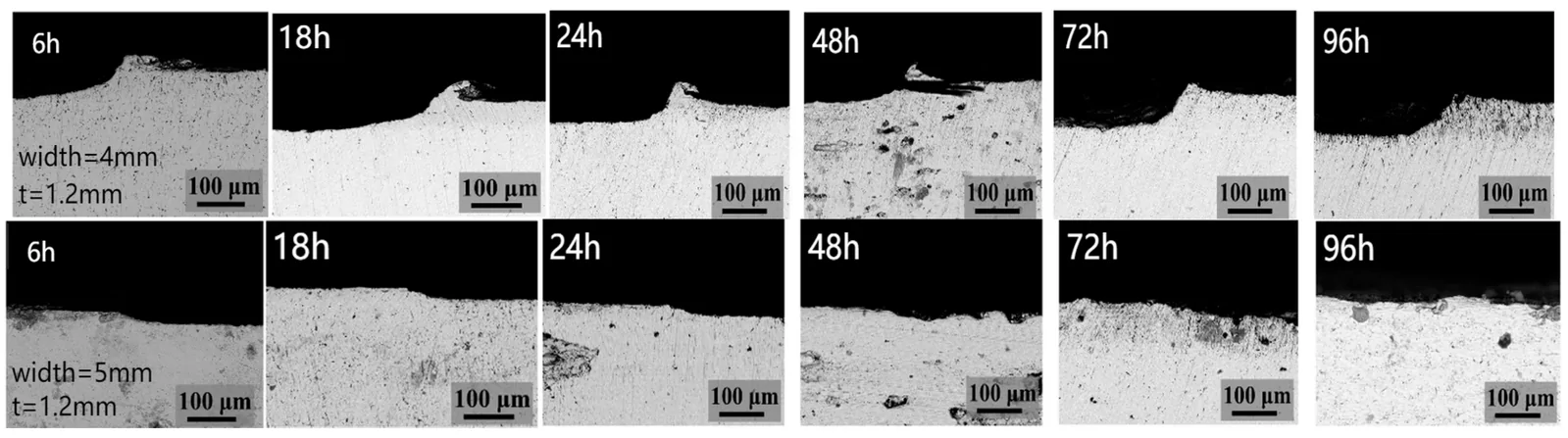
SEM images of the 4 mm and 5 mm scratch-width specimens (t = 1.2 mm) after different NSS exposure durations.

At 4 and 5 mm (Figure 9), both the depth and lateral extent of substrate corrosion exceed those at 3 mm. The enlarged cathodic area and longer electrolyte path reduce the share of zinc-derived protective current reaching the center, allowing steel dissolution to dominate increasingly large parts of the scratch.

The cross-sectional sequence separates the numerical and experimental roles. The 1, 3, and 5 mm model fields explain why protection decreases as scratch width increases. The experimental corrosion-depth results compare the 1–5 mm NSS conditions: corrosion remains comparatively limited through 2 mm and changes markedly at 3 mm. Thus, the change between the sampled 2 and 3 mm conditions is an experimental observation, while 3 mm is the first sampled condition showing pronounced protection loss rather than a precisely calculated or statistically established threshold.

#### 4.2.2. Corrosion-Product Analysis

**Figure 10:**
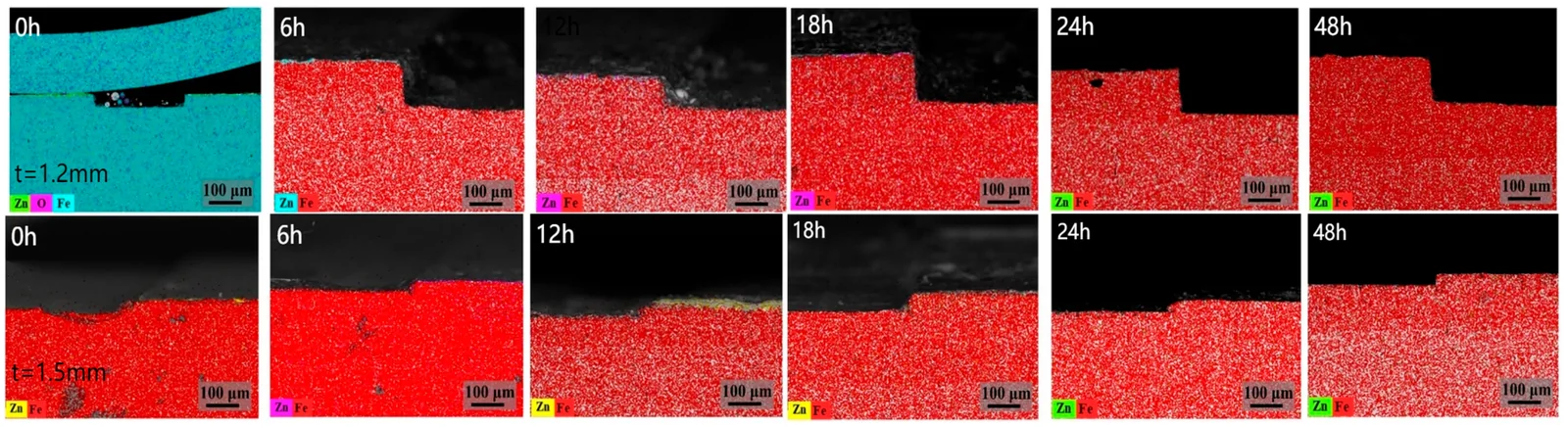
EDS analysis of the 1 mm scratch-width specimens (t = 1.2/1.5 mm) after different NSS exposure durations.

Cross-sectional EDS results after different NSS exposure durations are shown in Figure 10. Zinc-rich deposits appear on the exposed-steel region while zinc at the scratch edges is consumed, which is qualitatively consistent with the proposed sequence of zinc dissolution, Zn^2+^ transport, and zinc-containing product deposition. The two sheet gauges show similar qualitative elemental redistribution. These observations are used as descriptive corroboration and do not establish reaction rates, product quantities, or quantitative numerical accuracy.

**Figure 11:**
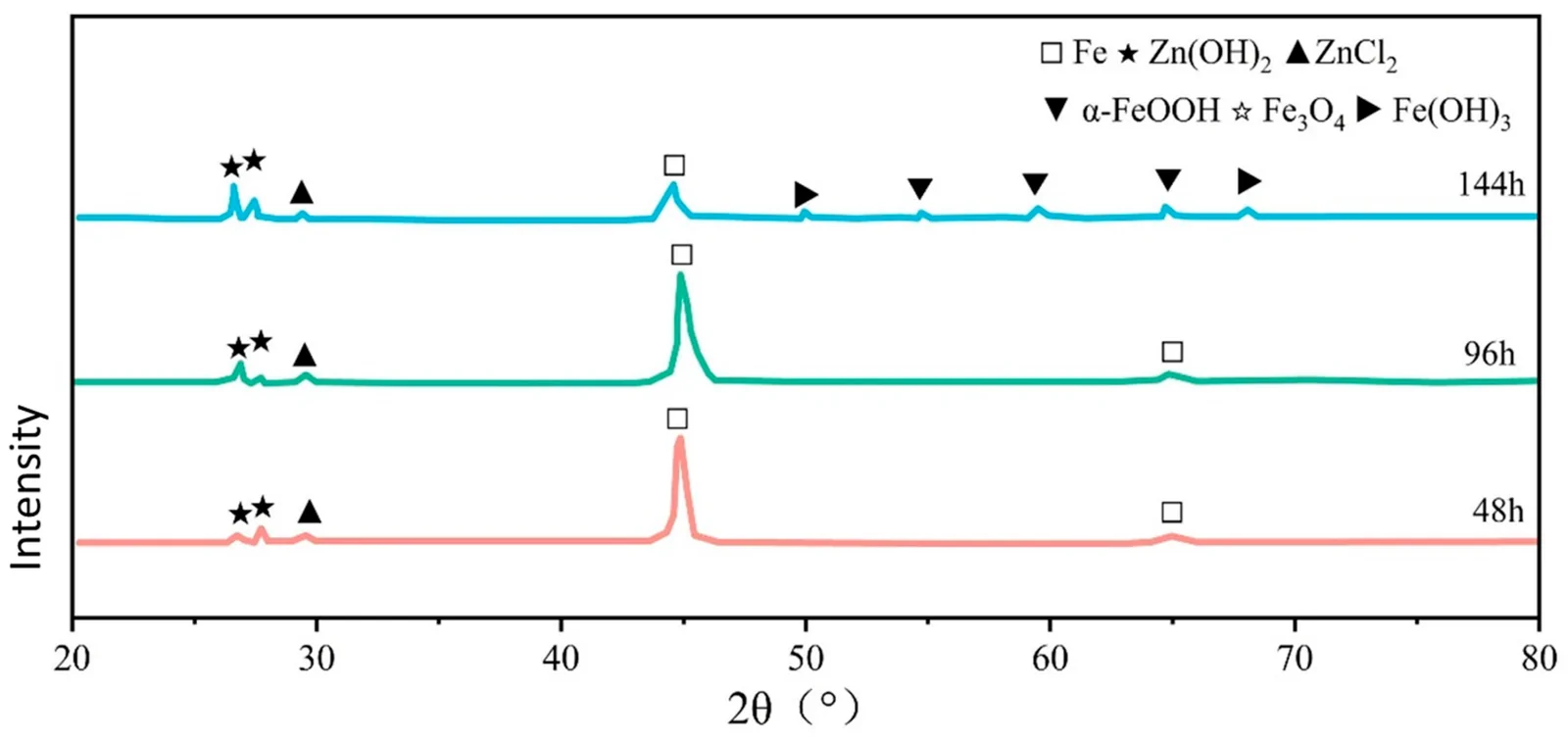
XRD patterns of corrosion products on the 1 mm scratch-width specimen after different NSS exposure durations. The Fe_3_O_4_ label represents a tentative phase assignment and is not used in the mechanistic interpretation.

Figure 11 shows the XRD patterns of corrosion products on the 1 mm scratched specimens after 48, 96, and 144 h of NSS exposure. Peaks were assigned to Zn(OH)_2_ and ZnCl_2_ following the phase assignments adopted in Ref. [25]. With increasing exposure time, α-FeOOH and Fe(OH)_3_ become more prominent, indicating a growing contribution from steel corrosion as zinc is consumed and product coverage becomes less effective. A Fe_3_O_4_ label is also present in the plotted phase assignment; because the retained record does not include independent peak fitting or reference-card analysis, it is treated as tentative and is not used as a basis for the mechanism. The phase evolution is consistent with the literature on zinc-product formation and protective coverage [11,17], but it is not treated as proof of complete product chemistry in the numerical model.

#### 4.2.3. Corrosion-Depth Trends

**Figure 12:**
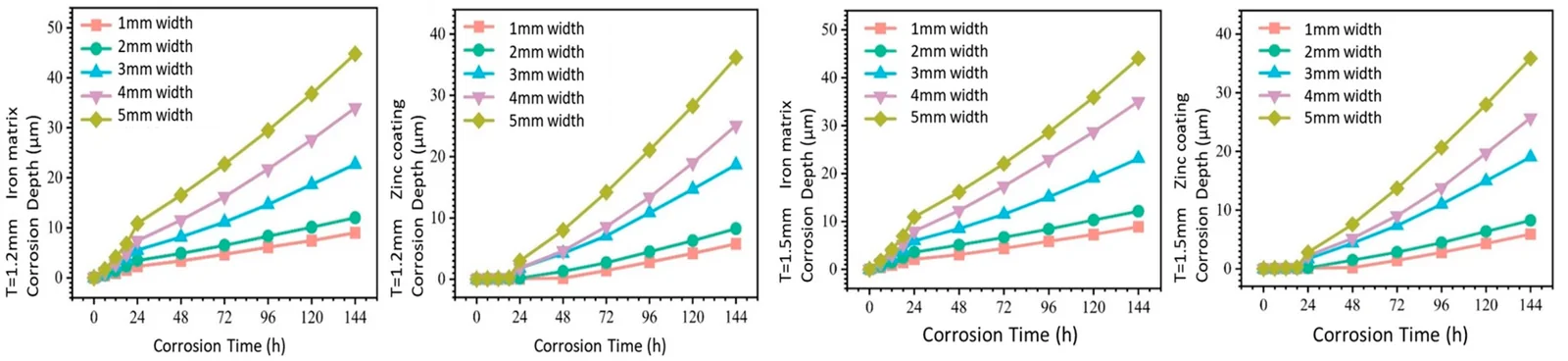
Corrosion depth as a function of exposure time for specimens with different scratch widths under NSS exposure. The curves are descriptive; replicate-level uncertainty estimates and statistical tests are not available. The comparison presents data up to 144 h.

Figure 12 shows the corrosion-depth evolution for the 1, 2, 3, 4, and 5 mm NSS conditions. The curves do not contain replicate-level uncertainty information and are therefore presented without error bars or inferential statistical testing. For both 1.2 and 1.5 mm sheets, the 1 and 2 mm conditions show comparatively limited corrosion, whereas an apparent marked change is observed between the sampled 2 and 3 mm conditions. The 3 mm condition is the first sampled width showing pronounced protection loss; this wording denotes descriptive separation among the sampled conditions, not a statistically established discontinuity or universal threshold. The observations are qualitatively consistent with the numerical mechanism of greater cathodic demand and ohmic loss with increasing width, but they are not used for direct simulated-versus-measured depth validation. The comparison in Figure 12 presents data up to 144 h.

### 4.3. Mechanistic Discussion of Scratch-Width-Dependent Protection Loss

The scratch-width effect follows a continuous electrochemical chain. A wider scratch exposes more steel, increasing the cathodic area and the oxygen-reduction current required for protection. At the same time, the path from the zinc edges to the scratch center lengthens. Electrolyte resistance therefore produces a larger ohmic drop, and the protective current decays before reaching the center. Once the center can no longer be maintained at a sufficiently negative potential, Fe begins to dissolve locally and the damaged region shifts from zinc-supported protection toward self-corrosion. The 1, 3, and 5 mm numerical field cases illustrate this proposed mechanism, while the experimental corrosion-depth results provide a descriptive comparison of the 1–5 mm NSS conditions. The apparent marked change occurs between the sampled 2 and 3 mm conditions, with 3 mm being the first sampled condition showing pronounced protection loss rather than a statistically established discontinuity or transferable maintenance criterion.

The two project-supplied sheet gauges show broadly similar protection-loss behavior under NSS. Any modest difference in propagation rate is treated as a secondary descriptive observation. The behavior is interpreted in terms of local zinc–steel geometry, cathode/anode area ratio, oxygen reduction, interfacial kinetics, electrolyte resistance, and corrosion-product coverage rather than bulk sheet volume or heat capacity.

This interpretation is consistent with thin-electrolyte and cut-edge studies showing that zinc protection is limited by cathode/anode geometry, electrolyte resistance, oxygen supply, and product coverage [5,6,7,17,26]. In the numerical model, corrosion-product effects are included through a simplified deposition and transport-resistance treatment. This functional component contributes to redistribution of concentration, potential, and current without implying a fully reconstructed porous-medium closure model.

### 4.4. Applicability and Model Limitations

The model is two-dimensional, assumes a stationary continuous electrolyte film and idealized scratch geometry, and treats the intact polymer-coated region as perfectly insulating. It uses an enlarged 100 µm computational zinc domain rather than the measured 8 ± 2 µm specimen coating. This choice changes modeled zinc inventory and local anode/cathode geometry, and no zinc-thickness sensitivity study is available. Corrosion-product effects are represented through a simplified functional deposition/transport-resistance treatment. Three-dimensional morphology, stochastic pitting, droplet breakup, complete wet–dry cycling, local delamination, and product-layer cracking are not resolved. The absence of a mesh-independence study and complete specification of the reaction-specific Nernst terms, oxygen boundary condition, product-resistance formulation, experimental reference electrode, solver, time stepping, and convergence criteria prevents strict independent numerical reproduction. The results therefore support only mechanism-oriented interpretation and scratch-width trend comparison, not quantitative potential, current, corrosion-depth, service-life, or repair-limit prediction.

The experimental dataset does not contain replicate-level corrosion-depth data sufficient for uncertainty analysis or statistical testing, and it does not include EIS, fitted impedance parameters, or independent electrochemical potential/current measurements. SEM, EDS, and profilometry are therefore used as qualitative or descriptive corroboration rather than quantitative validation. Replicated corrosion-depth measurements, electrochemical testing, and direct model-to-experiment calibration are left for future work.

## 5. Conclusions

A through-scratch forms a Zn-anode/Fe-cathode galvanic couple. Increasing scratch width enlarges the exposed-steel cathodic area and its oxygen-reduction demand while lengthening the ionic-current path from the zinc edges to the center.

Electrolyte resistance produces an increasing ohmic drop across the damaged region, so the protective current reaching the center decreases with width and local Fe dissolution begins when the center can no longer be maintained at a sufficiently negative protective potential. The numerical 1, 3, and 5 mm cases explain this mechanism; the experimental 1–5 mm NSS results show a marked change between the sampled 2 and 3 mm conditions, with 3 mm serving only as the first sampled condition showing pronounced protection loss.

The model includes a simplified corrosion-product deposition and transport-resistance treatment that contributes to field redistribution. It supports mechanism-oriented interpretation without claiming a fully reconstructed porous-medium model, independent product-layer calibration, one-to-one corrosion-depth prediction, or quantitative electrochemical validation. The 100 µm computational zinc domain is not specimen-scale and was not subjected to a thickness-sensitivity study; consequently, absolute current, interface recession, coating life, and a universal critical width are not claimed.

## Figures and Tables

**Table 1:** Electrochemical reaction network considered in the model and interpretation.

Process	Reaction/Model Treatment	n and Standard Potential, E^0^	Baseline Inclusion and Physical Role
Zinc dissolution	Zn → Zn^2+^ + 2e^−^	n = 2; E^0^ = −0.763 V vs. SHE	Included. Primary anodic reaction; supplies electrons and the Zn^2+^ boundary flux.
Oxygen reduction	O_2_ + 2H_2_O + 4e^−^ → 4OH^−^	n = 4; E^0^ = +0.401 V vs. SHE	Included for NSS. Main cathodic path on exposed steel; couples O_2_ consumption to OH^−^ generation.
Product deposition and dissolution	Zn^2+^ + 2OH^−^ ⇌ Zn(OH)_2_(s)	—	Included through a simplified deposition/transport-resistance treatment.
Iron dissolution	Fe → Fe^2+^ + 2e^−^	n = 2; E^0^ = −0.440 V vs. SHE	Local iron dissolution is introduced when the steel becomes insufficiently protected; Fe^2+^ transport and its concentration-dependent equilibrium-potential correction are outside the mechanism-oriented formulation.
Supporting-ion transport	Nernst–Planck transport of Na^+^ and Cl^−^	z_Na_ = +1; z_Cl_ = −1	Included. Carries ionic current and represents chloride migration/diffusion without assigning Cl^−^ an electron-transfer reaction.

**Table 2:** (**a**) Geometry, exposure, and transport inputs used in the numerical model. (**b**) Kinetic, material, and boundary-condition inputs used in the numerical model.

Parameter	Value	Unit	Status and Basis	Physical Role
(**a**)				
Model domain	2D scratched HDG steel/electrolyte cross-section	-	Idealized computational domain used for comparative analysis	Represents the local through-scratch Zn-steel-electrolyte couple.
Computational zinc-domain thickness, δZn	100	µm	Enlarged numerical domain; not experimentally calibrated	Keeps the moving zinc domain resolvable; changes modeled zinc inventory and is excluded from quantitative comparison.
Simulated scratch width, w	1, 3, 5	mm	Model input used for qualitative comparison	Changes exposed-steel demand and the ionic path from zinc edges to the center.
Experimental scratch width, w_exp_	0.5, 1, 2, 3, 4, 5	mm	Experimental groups used in the present study	Provides discrete observations for trend-level comparison.
NSS NaCl concentration	5	wt.%	GB/T 10125-2021 [24]	Defines the near-neutral supporting electrolyte.
Nominal Na^+^ and Cl^−^ bulk concentration	≈856 each	mol m^−3^	Derived model input from 5 wt.% NaCl idealization	Sets the far-field supporting-ion concentrations.
NSS pH	6.5–7.2	-	GB/T 10125-2021 [24]; documented test condition	Defines the near-neutral exposure chemistry.
NSS temperature, T	308.15 (35 ± 1 °C)	K	GB/T 10125-2021 [24]; documented test condition	Enters the kinetic and migration terms.
Mobile species	Zn^2+^, OH^−^, Na^+^, Cl^−^, O_2_(aq)	-	Documented model configuration	Connects interfacial reactions to electrolyte transport.
Species charge numbers, z_i_	+2, −1, +1, −1, 0	-	Reaction/charge stoichiometry	Controls electromigration; dissolved O_2_ is neutral.
Zn^2+^ diffusion coefficient	0.70 × 10^−9^	m^2^ s^−1^	Model input used for qualitative comparison	Controls spreading of anodically generated Zn^2+^.
OH^−^ diffusion coefficient	5.27 × 10^−9^	m^2^ s^−1^	Model input used for qualitative comparison	Controls transport of cathodically generated OH^−^.
Na^+^ diffusion coefficient	1.33 × 10^−9^	m^2^ s^−1^	Model input used for qualitative comparison	Represents supporting-cation transport.
Cl^−^ diffusion coefficient	2.03 × 10^−9^	m^2^ s^−1^	Model input used for qualitative comparison	Controls chloride diffusion and electromigration.
Dissolved-O_2_ diffusion coefficient	2.0 × 10^−9^	m^2^ s^−1^	Model input used for qualitative comparison	Controls oxygen supply to the neutral cathodic reaction.
Oxygen-supply condition	Common boundary condition applied to all cases	—	Not independently calibrated to NSS specimens	Provides a common oxygen supply for qualitative comparison among scratch widths.
Effective electrolyte conductivity, κ	8.0	S/m	Model input used for qualitative comparison	Controls electrolyte-potential gradients and ohmic loss.
Corrosion-product treatment	Simplified functional deposition and transport resistance	—	Simplified functional treatment; not independently calibrated	Represents qualitative field redistribution only.
Electrolyte velocity, u	0	m/s	Static-electrolyte assumption adopted in the present model	Eliminates convection; transport comprises diffusion and electromigration.
Mesh	Free triangular; local interface refinement	—	No separate mesh-independence study	Supports qualitative field interpretation, not mesh-verified absolute prediction.
(**b**)				
Faraday constant, F	96,485	C mol^−1^	Fundamental physical constant	Converts current to molar flux and interface velocity.
Universal gas constant, R	8.314	J mol^−1^ K^−1^	Fundamental physical constant	Appears in Butler–Volmer and Nernst–Planck terms.
Transferred electrons, n	Zn: 2; Fe: 2; O_2_: 4	-	Reaction stoichiometry	Sets current-to-molar-rate conversion.
Charge-transfer coefficients, α_a_ and α_c_	0.5, 0.5	-	Model input used for qualitative comparison	Controls Butler–Volmer sensitivity to overpotential.
Reaction standard potentials, E^0^	Zn: −0.763; Fe: −0.440; O_2_/OH^−^: +0.401	V vs. SHE	Standard-potential inputs adopted in the documented model configuration	Define the reference states used to calculate local E_e_q.
Equilibrium-potential treatment	Nernst framework	—	Generalized framework used for comparative interpretation	Relates the interfacial equilibrium potential to the local electrochemical state.
Zn exchange current density	1.0 × 10^−2^	A m^−2^	Model input used for qualitative comparison	Sets the zinc charge-transfer scale.
Fe exchange current density	1.0 × 10^−2^	A m^−2^	Model input used for qualitative comparison	Sets the iron charge-transfer scale where steel becomes anodic.
O_2_-reduction exchange current density	1.0 × 10^−4^	A m^−2^	Model input used for qualitative comparison	Sets the oxygen-reduction kinetic scale.
Zinc molar mass, M_Zn_	65.38	g mol^−1^	Fundamental material property	Used in the Faradaic interface-velocity relation.
Zinc density, ρ_Zn_	7.14	g cm^−3^	Fundamental material property	Converts dissolved zinc amount to geometric recession.
Iron molar mass, M_Fe_	55.85	g mol^−1^	Fundamental material property	Used if steel-interface recession is evaluated.
Iron density, ρ_Fe_	7.87	g cm^−3^	Fundamental material property	Converts dissolved iron amount to geometric recession.
Electrode current boundary	−n·i_l_ = j_loc_	A m^−2^	Reaction-derived boundary condition	Couples interfacial kinetics to electrolyte current.
Insulating boundary	n·i_l_ = 0	A m^−2^	Boundary condition adopted in the present model	Prevents ionic current through intact coated regions.
Electrolyte-potential reference condition	φl = 0 numerical datum; no experimental reference-electrode mapping	V	Numerical reference only	Defines the solution datum but does not support comparison with measured electrode potential.
Initial electrolyte state	Uniform supporting-ion concentrations listed in Table 2a; φl = 0	—; V	Initial condition used in the model	Initializes uniform species concentrations and the electrolyte-potential datum.
Open concentration boundary	c_i_ = c_i,bulk_	mol m^−3^	Boundary condition used in the model	Maintains the assigned supporting-species bulk condition.
Zn^2+^ flux at zinc	−n·N_Zn_^2+^ = j_a_/(2F)	mol m^−2^ s^−1^	Faraday-law boundary condition	Converts zinc anodic current into a Zn^2+^ source.
O_2_ and OH^−^ fluxes at steel	−n·N_O2_ = −j_c_/(4F); −n·N_OH_^−^ = j_c_/F	mol m^−2^ s^−1^	Oxygen-reduction stoichiometry	Consumes O_2_ and generates OH^−^ on cathodic steel.
Solver, time stepping, and convergence	Complete settings not reported for independent reproduction	—	Reproducibility limitation	Prevents strict independent numerical reconstruction and absolute-value verification.

**Table 3:** (**a**) Chemical composition of the steel substrate (wt.%). (**b**) Measured zinc-coating mass of the Z60/60 product.

(**a**)						
**C**	**Si**	**Mn**	**P**	**S**	**Ti**	**Fe**
0.015	0	0.20	0.011	0.009	0	Balance
(**b**)						
**Total Coating Mass (g/m^2^)**		**Top-Side Coating Mass (g/m^2^)**			**Bottom-Side Coating Mass (g/m^2^)**	
122		61			61	

Note: (a) Values are mass fractions in wt.%; Fe is the balance; (b) The nominal Z60/60 designation corresponds to approximately 60 g/m^2^ per side; the measured values were 61 g/m^2^ per side and 122 g/m^2^ in total.

## Data Availability

The original contributions presented in this study are included in the article. Further inquiries can be directed to the corresponding author.
